# Liraglutide Increases Serum Levels of MicroRNA-27b, -130a and -210 in Patients with Type 2 Diabetes Mellitus: A Novel Epigenetic Effect

**DOI:** 10.3390/metabo10100391

**Published:** 2020-09-30

**Authors:** Rosaria Vincenza Giglio, Dragana Nikolic, Giovanni Li Volti, Anca Pantea Stoian, Yajnavalka Banerjee, Antonio Magan-Fernandez, Giuseppa Castellino, Angelo Maria Patti, Roberta Chianetta, Carlo Castruccio Castracani, Giuseppe Montalto, Ali A. Rizvi, Giorgio Sesti, Manfredi Rizzo

**Affiliations:** 1 Department of Health Promotion Sciences Maternal and Infantile Care, Internal Medicine and Medical Specialties (PROMISE), University of Palermo, 90127 Palermo, Italy; rosaria.vincenza.giglio@alice.it (R.V.G.); dragana.nikolic@unipa.it (D.N.); amaganf@ugr.es (A.M.-F.); castellinogiusy@gmail.com (G.C.); pattiangelomaria@gmail.com (A.M.P.); chianetta.roberta8@gmail.com (R.C.); giuseppe.montalto@unipa.it (G.M.); manfredi.rizzo@unipa.it (M.R.); 2 Department of Biomedical and Biotechnological Sciences, University of Catania, 95125 Catania, Italy; livolti@unict.it (G.L.V.); carlo.castruccio@unict.it (C.C.C.); 3 Department of Diabetes, Nutrition and Metabolic Diseases, Carol Davila University of Medicine and Pharmacy, 050474 Bucharest, Romania; ancastoian@yahoo.com; 4 College of Medicine, Mohammed Bin Rashid University of Medicine and Health Sciences, Dubai, UAE; Yajnavalka.Banerjee@mbru.ac.ae; 5 Division of Endocrinology, Diabetes and Metabolism, University of South Carolina School of Medicine, Columbia, SC 29203, USA; 6 Division of Endocrinology, Metabolism, and Lipids Emory University School of Medicine, Atlanta, GA 30322, USA; 7 Department of Clinical and Molecular Medicine, University of Rome La Sapienza, 00182 Rome, Italy; giorgio.sesti@uniroma1.it

**Keywords:** liraglutide, microRNAs, type-2 diabetes, cardiometabolic risk, epigenetic

## Abstract

Liraglutide has shown favourable effects on several cardiometabolic risk factors, beyond glucose control. MicroRNAs (miRNAs) regulate gene expression, resulting in post-transcriptional modifications of cell response and function. Specific miRNAs, including miRNA-27b, miRNA-130a, and miRNA-210, play a role in cardiometabolic disease. We aimed to determine the effect of liraglutide on the serum levels of miRNA-27b, miRNA-130a and miRNA-210. Twenty-five subjects with type-2 diabetes mellitus (T2DM), naïve to incretin-based therapy, were treated with liraglutide (1.2 mg/day as an add-on to metformin) for 4 months. miRNAs were quantified using real-time polymerase chain reaction. After liraglutide treatment, we found significant reductions in fasting glucose (from 9.8 ± 5.3 to 6.7 ± 1.6 mmol/L, *p* = 0.0042), glycosylated haemoglobin (HbA1c) (from 8.1 ± 0.8 to 6.6 ± 1.0%, *p* = 0.0008), total cholesterol (from 5.0 ± 1.0 to 4.0 ± 0.7 mmol/L, *p* = 0.0011), triglycerides (from 1.9 ± 1.0 to 1.5 ± 0.8 mmol/L, *p* = 0.0104) and low-density lipoprotein cholesterol (from 2.9 ± 1.2 to 2.2 ± 0.6 mmol/L, *p* = 0.0125), while the serum levels of miRNA-27b, miRNA-130a and miRNA-210a were significantly increased (median (interquartile range, IQR) changes: 1.73 (7.12) (*p* = 0.0401), 1.91 (3.64) (*p* = 0.0401) and 2.09 (11.0) (*p* = 0.0486), respectively). Since the changes in miRNAs were independent of changes in all the metabolic parameters investigated, liraglutide seems to exert a direct epigenetic effect in T2DM patients, regulating microRNAs involved in the maintenance of endothelial cell homeostasis. These changes might be implicated in liraglutide’s benefits and may represent useful targets for cardiometabolic management.

## 1. Introduction

Liraglutide is a glucagon-like peptide-1 receptor agonist (GLP-1 RA) suitable for once-daily administration in patients with type-2 diabetes mellitus (T2DM). Liraglutide possesses glucose-lowering effects with a low risk of hypoglycaemic events and has a number of noteworthy non-glycaemic properties, including effects on body weight, plasma lipids, inflammatory markers and blood pressure, but also reduces oxidative stress, endothelial dysfunction and subclinical atherosclerosis and, consequently, ameliorates non-alcoholic fatty liver disease and non-alcoholic steatohepatitis and prevents cardiovascular (CV) events [1,2,3,4].

MicroRNAs (miRNAs) are endogenous, small non-coding RNA molecules (containing from 19 to 23 nucleotides) that participate in RNA silencing and the post-transcriptional regulation of gene expression. MiRNAs play important roles as regulators in developmental timing, apoptosis, cell proliferation, haematopoiesis, the patterning of the nervous system, cellular homeostasis, vascular inflammation and metabolism [5,6,7,8]. Accumulating evidence indicates that targeting the levels of specific miRNAs may successfully prevent or treat diabetes and its complications [9] as well as CV diseases, miRNAs being significant regulators and micro-managers of key cellular and molecular pathophysiological processes involved in CV disease [10]. However, innovative strategies are still needed to achieve the delivery of pharmacological tools that can efficiently modulate the levels of miRNAs in the appropriate target cells before these tools can be allowed for the treatment of diabetes [9].

Native GLP-1 was also shown to exert its actions through miRNAs in vitro, with an effect on insulin secretion mediated by miRNA-132 and miRNA-212 [11], and an increased anti-oxidant and anti-apoptosis activity mediated by miRNA-23a [12]. Similarly, GLP-1 decreases levels of miRNA-338, increasing β-cell function, followed by an improvement of diabetic conditions, and modulates some miRNAs’ expression in the liver, regulating hepatic lipid storage: it downregulates miRNA-34a and miRNA-21 and upregulates miRNA-200b and miRNA-200c [13].

The serum levels of miRNA-130a, miRNA-27b and miRNA-210 represent potential biomarkers for early-stage arteriosclerosis [14] and acute myocardial infarction [15]. Furthermore, they may serve as useful markers not only for the diagnosis of CV disease but also for disease progression, risk stratification and future therapeutic interventions [16]. In particular, miRNA-27b is a central upstream regulator of the transcriptional network involved in beige and brown adipogenesis [17]. The activities of brown and beige fat cells are involved in metabolic disorders since they can influence the type and degree of obesity and its impact on CV risk [18,19]. Furthermore, miRNA-27b has important roles in endothelial–mesenchymal transition [20], and decreased miRNA-27b expression was reported to reduce the angiogenic activity of endothelial cells in atherosclerotic mice [21]. Similarly, the downregulation of miRNA-130a may be an underlying mechanism of endothelial dysfunction in T2DM [22] as well as being related with the development of T2DM [23], while in a model of heart failure, miRNA-130a improved cardiac function [24]. In addition, miRNA-130a may have therapeutic potential in coronary heart disease (CHD) [25] since its levels are decreased in CHD patients compared with controls, thus potentially predicting CV risk [26]. Finally, miRNA-210 serves as a possible regulator of oxidative stress and can act as a hypoxia-inducible factor (HIF) [27], thus playing a role in clinical conditions that affect morbidity and mortality, including cardiac and peripheral ischemia, inhibiting apoptosis and regulating cell proliferation, differentiation, migration, mitochondrial metabolism and angiogenesis in hypoxic cells [28].

The evidence regarding the impact of liraglutide treatment on miRNAs is scarce, limited to a few studies published in animal models. In diabetic rats, liraglutide reduced pancreatic β-cell apoptosis in association with a 90% decrease in miRNA-139-5p expression [29] and significantly ameliorated blood glucose, insulin resistance status and endothelial function through miRNAs [30]. In addition, liraglutide can improve the viability of pancreatic β-cells and increase pancreatic α-cell apoptosis via modifications in miRNA-375 levels in murine cell lines in vitro [31]. However, the effect of liraglutide treatment on miRNA-27b, miRNA-130a and miRNA-210 and its impact on cardiometabolic diseases [7] in humans is still unknown. Clinical studies are, therefore, warranted in order to provide novel diagnostic and therapeutic approaches for cardiometabolic risk management in T2DM patients. Thus, the present study aimed to determine the impact of liraglutide treatment on the serum levels of miRNA-130a, miRNA-27b and miRNA-210 in a 4-month follow-up study.

## 2. Results

As shown in Table 1, 25 T2DM patients were included in the study (mean age = 64.6 ± 8.4 years; 36% women; median HbA1c = 8.3%; mean diabetes duration = 9.6 ± 7.1 years). Fifty-two percent of the patients had a duration of diseases higher than 9 years. All patients were treated with metformin only, but in some of them, sulfonylurea use was part of their medical history of diabetes treatment (data not shown). Forty percent of the patients were current smokers, 72% had hypertension, 64% were obese, and 76% had dyslipidaemia.

Table 2 shows the changes observed in cardiometabolic risk parameters. After 4 months of liraglutide therapy, significant reductions in fasting glucose (*p* = 0.0052), HbA1c (*p* = 0.0009), total cholesterol (*p* = 0.0007), triglycerides (*p* = 0.0478) and LDL-C (*p* = 0.0150) were observed. Blood pressure and heart rate did not change significantly after therapy (data not shown).

**Figure 1:**
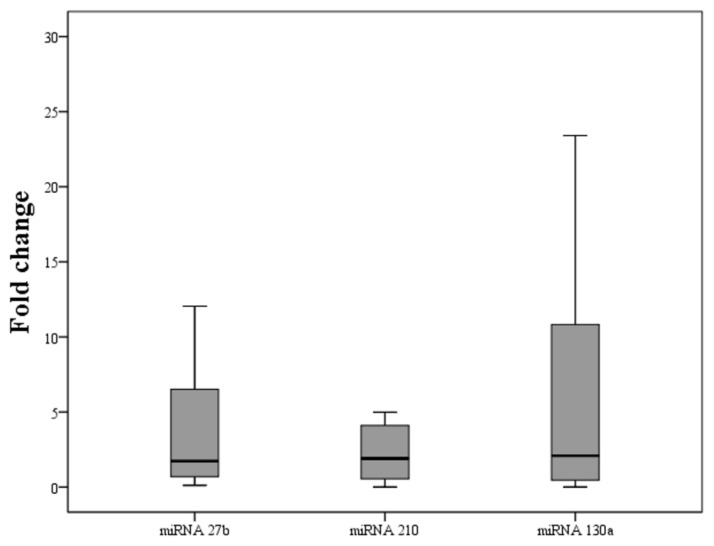
Changes in serum levels of miRNAs after 4 months of liraglutide therapy.

Furthermore, as shown in Table 3 and Figure 1, serum miRNA-27b, miRNA-130a and miRNA-210 levels were significantly increased after liraglutide treatment (*p* = 0.0401, *p* = 0.0401 and *p* = 0.0486, respectively).

Correlation analysis revealed that changes in miRNAs were not associated with changes in any of the metabolic parameters investigated (Table 4). Multiple regression analysis revealed that there were no significant independent predictors of changes for specific miRNAs (data not shown).

## 3. Discussion

In the present study, 4 months of therapy with liraglutide in T2DM patients significantly improved a number of cardiometabolic risk factors, including fasting glucose, HbA1c, total cholesterol, triglycerides and low-density lipoprotein cholesterol (LDL-C), while the reductions in body weight and body mass index (BMI) approached, but did not reach, statistical significance. Furthermore, the serum levels of miRNA-130a, miRNA-27b and miRNA-210 were significantly increased during the study. To our knowledge, this is the first in vivo study in humans showing an effect of liraglutide on miRNAs.

T2DM is a major risk factor for CV disease; both of these disorders are associated with endothelial dysfunction and vascular complications [32,33]. MiRNAs have been linked to T2DM since Zampetaki et al. [34] first reported a specific miRNA feature in T2DM patients characterised by a decrease in endothelial miRNA-126. This mechanism could explain the impaired angiogenesis observed in T2DM patients. Afterwards, it has been shown that miRNAs play a role in the development of long-term T2DM complications [13]. The early use of metformin, together with lifestyle interventions (dietary modification, increased physical activity and weight loss), represents the initial treatment of T2DM [35]. However, the majority of T2DM patients cannot achieve HbA1c targets with this therapeutic approach, and thus, the use of other antidiabetic drugs is often required. In this context, the American Diabetes Association (ADA)/the European Association for the Study of Diabetes (EASD) recommended the use of GLP-1 RA as the first-line injectable in such T2DM subjects who do not achieve HbA1c targets with one or more oral glucose-lowering agents. Liraglutide has also shown better efficacy, with proper safety, in relation to other GLP-1 RAs, such as lixisenatide [36]. We need to emphasize that the importance of a correct lifestyle is always crucial [37], and in recent years, several natural approaches in metabolic syndrome management have been found to be beneficial [38,39].

Liraglutide can be used as a second-line antidiabetic agent, and it has been associated with cardiometabolic risk reduction independently of its glucose-lowering effects [40,41]. As widely known, the Liraglutide Effect and Action in Diabetes: Evaluation of Cardiovascular Outcome Results (LEADER) trial, published in 2016, reported that liraglutide significantly decreased the composite of the occurrence of nonfatal myocardial infarction, nonfatal stroke or CV death as well as all-cause mortality and the composite outcome of retinal or renal events in T2DM patients [42]. Furthermore, liraglutide exerts anti-oxidative, anti-apoptotic and anti-inflammatory properties [43,44], and these effects could be mediated by its impact on miRNA levels [29,45,46] which is in accordance with our findings. Furthermore, it seems that liraglutide may protect the myocardium against ischemia/reperfusion injury, possibly through reducing cardiomyocyte apoptosis and oxidation [47]. Therefore, the modulation of miRNA expression might contribute to the reduction in CV risk observed in T2DM patients treated with liraglutide [48,49]. It is noteworthy that other miRNAs different from the ones reported in this study have also been identified as biomarkers of early-stage diabetic atherosclerosis, such as miRNA-21, miRNA-218, miRNA-211 and miRNA-126 [50,51]. Those that were measured in the present study, miRNA-130a, miRNA-27b and miRNA-210, have been previously related to endothelial cell homeostasis, vascular inflammation and metabolic disorders [16].

Prior studies reported that miRNA-27b is involved in the regulation of adipogenesis [17]. For example, miRNA-27b produced an anti-adipogenic effect by inhibiting prohibitin in an in vitro model of adipose-derived stem cells [52]. miRNA-27b was also shown to affect endothelial cells, being the second miRNA described as critical for angiogenesis along with miRNA-126 [53]. In this context, a decreased expression of miRNA-27b was related to lower angiogenic activity and neovascularisation through the gene Naa15 in the endothelium of atherosclerotic mice [21]. However, it should be highlighted that the last study was performed in the condition of the genetic deficiency of CCR6, and it remains unknown how CCR6 loss contributes to the regulation of miRNA-27b.

Furthermore, miRNA-27b inhibited the expression of monocyte chemoattractant protein-1 stimulated by interleukin-17 in cardiomyocytes, thus highlighting its potential use in mitigating myocardial injury [54]. Moreover, it has also been suggested as a novel therapeutic target for the treatment of cardiac dysfunction, having an anti-fibrotic role in the left atrium and its function [55]. miRNA-27b may play a role in plasma lipid regulation through genes such as angiopoietin-like-3 and glycerol-3-phosphate acyltransferase-1; its expression was altered in the presence of hyperlipidaemia and atherosclerosis in a murine model [56]. It has been suggested that liraglutide may inhibit proliferation and promote apoptosis in human breast cancer cells through the inhibition of miR27a expression [57], providing an experimental basis for treatment strategies for T2DM patients with breast cancer. However, only 100 nM liraglutide inhibited miR-27a expression, while 10 nM liraglutide did not lead to a statistically significant decline. Thus, further investigation is required, and specific attention should be paid to the different clinical conditions and mechanisms involved.

miRNA-130a has been associated with T2DM; its expression was downregulated in epithelial progenitor cells from T2DM patients, leading to the activation of mitogen-activated protein 3K12 and the c-Jun N-terminal kinase pathway [22]. These pathways are involved in insulin resistance, atherosclerosis, apoptosis and the impairment of epithelial cell function [22]. However, this effect was not observed in pancreatic β-cells, where miRNA-130a overexpression was associated with lower ATP levels and an impaired function of β-cells [58]. miRNA-130a has also been linked to obesity; chronic nutritional stress and overfeeding can induce the expression of miRNAs known to regulate lipid and glucose metabolism, potentially modulating inflammation [59]. Decreased circulating levels of miRNA-130a have also been found in CHD patients [26].

We found in the present study that liraglutide beneficially modulated serum levels of miRNA-130a, and, in the long-term, such an effect may exert a hepatoprotective effect. Zheng et al. have shown that miRNA-130a targets the 3’-untranslated region of Rho-kinase 2, inhibiting the proliferation, migration and invasive ability of hepatocellular carcinoma cells [60]. This topic deserves further investigation, because some evidence even suggests that T2DM could increase the risk of hepatocellular carcinoma per se [61]. Furthermore, miRNA-130a may increase the radiosensitivity of rectal cancer cells by directly targeting SOX4 [62]; since T2DM has been associated with a 1.3-fold increased risk of colorectal cancer, further research in this field is warranted too [63]. Additionally, microRNA-130a, cooperatively with microRNA-145, through the inactivation of Runt-related transcription factor 3, increases cell proliferation and tumour angiogenesis in gastric cancer [64]; since T2DM increases the risk of gastric cancer following *Helicobacter pylori* eradication [65], this represents another field that deserves further investigation.

miRNA-210 is involved in oxidative stress regulation, glucose metabolism and apoptosis, partly through hypoxia, a key component in CV disease, acting as a HIF [27]. miRNA-210 was also shown to be a pleiotropic mediator in angiogenesis, affecting cell survival, migration and differentiation, as well as modulating the endothelial cell in vitro response to hypoxia [66]. In this context, miRNA-210 may exert cytoprotective activity in the ischemic skeletal muscle, regulating oxidative stress and metabolism [67]. In addition, miRNA-210 may contribute to the stabilisation of carotid plaques by the inhibition of adenomatous polyposis coli expression, affecting Wingless-related integration site (Wnt) signalling and regulating smooth muscle cell survival [68]. Furthermore, in vitro studies demonstrated a cardioprotective role of miRNA-210 in H_2_O_2_-induced cardiomyocyte apoptosis by regulating the pro-apoptotic Bcl-2 adenovirus E1B 19kDa-interacting protein 3 (BNIP3) [69], as well as it being implicated in the mechanisms of hypoxic-mesenchymal-stem-cell-derived exosomes for cardiac repair after myocardial infarction [70]. Such findings are in accordance with the favourable changes in oxidative stress seen after liraglutide treatment in a large number of preclinical and clinical studies [71]. Strong evidence suggests miRNAs as promising diagnostic biomarkers and therapeutic targets in several diseases, including CV disease and T2DM [72,73,74].

miRNA-210 also targets the ephrin-A3 (Efna3) and protein tyrosine phosphatase-1B (Ptp1b) genes, endogenously regulating angiogenesis and apoptosis, respectively [75]. Efna3 is a gene involved in the inhibition of angiogenesis, although the gene’s exact role is still not fully elucidated. miRNA-210 has been shown to quell Efna3 at the level of transcription, allowing angiogenesis to ensue in post-infarction cardiac tissue [75]. Ptp1b, on the other hand, is involved in the induction of apoptosis through the regulation of phosphorylation caspases 3 and 8 [76]. miRNA-210 has been shown to impede the effects of the Ptp1b protein, which suppresses its pro-apoptotic functions. Therefore, the suppression of these two particular genes may contribute to the improvement of cardiac tissue and function by upregulating angiogenesis and inhibiting the apoptosis of cardiomyocytes after myocardial infarction [75]. Future lines of investigation should consider the evaluation of the modulatory effects of liraglutide on these genes.

In the present study, liraglutide was shown to affect the serum levels of selected miRNAs in T2DM patients, together with improvements in several cardiometabolic risk factors. Further studies are needed to confirm this epigenetic effect of the regulation of miRNA expression by liraglutide as well as other GLP-1 RAs in patients with T2DM. Of note, a recent review proposes that miRNAs clearly play a significant role on T2DM and its related complications and that the evaluation of miRNAs may serve as new potential methods of the assessment of cardiometabolic risk, as well as new therapeutic targets for the treatment of both T2DM and CV disease [77]. Other studies in animal models have also shown that liraglutide may exert an effect on bone marrow-derived miRNAs and also may explain the potential protective effects of liraglutide on bone metabolism [78].

The present study has some potential limitations, such as the small sample, the relatively short duration of the research and the lack of a control group. However, our study also has several strengths that need to be clearly stated. First, this is a real-world study, evaluating, very carefully, the effects of liraglutide treatment in daily clinical practice. Second, the dietary habits of the participants remained unchanged, since specific nutrients or compounds might modulate the expression of miRNAs. Third, we did our best to avoid confounding factors, and therefore, all measurements were assessed in aliquots with blinded codes. Fourth, the compliance was very high, and, last, this study was performed spontaneously, independently of any pharmaceutical company.

Future studies with larger cohorts and a longer follow-up are required to establish the impact of liraglutide treatment, and GLP-1 RAs in general, on distinct miRNAs in T2DM patients. Such trials should also assess the associations, if any, between liraglutide-induced changes in miRNAs and cardiometabolic benefits.

## 4. Materials and Methods

Patients with T2DM from the Unit of Diabetes and Cardiovascular Prevention (University Hospital of Palermo, Italy) were enrolled consecutively in the present study. The inclusion criteria were age ≥18 years, treatment with metformin alone and naïve to incretin-based and obesity therapies. Participants were excluded if they had a history of renal failure, liver disease, CV events, cancer or severe infectious diseases such as human immunodeficiency virus (HIV), hepatitis B virus (HBV) or hepatitis C virus (HCV). The protocol was in accordance with the Helsinki Declaration of 1975 as revised in 2013, and it was approved by the Research Ethics Council of the University of Palermo. The present study is registered in clinicaltrials.gov (NCT01715428). Signed informed consent was obtained from all participants before entering the study.

Treatment with liraglutide (0.6 mg/day for 2 weeks, followed by 1.2 mg/day), administered subcutaneously, was added to metformin (1500 mg/day orally) at a maintained dosage for 4 months. We decided to use the daily dose of 1500 mg so that 500 mg of metformin was administered three times daily (at breakfast, lunch and dinner). All the other drugs remained unchanged throughout the study in order to minimize potential confounding effects. However, before enrolment in the present study, all the patients were on stable doses of concomitant drugs at least 4 weeks immediately prior to study entry. Since specific nutrients or bioactive compounds in the diet have been shown to exert a modulatory effect on the expression of miRNAs in the physiological milieu [79], special clinical care was taken to ensure that the dietary habits of the participants remained unchanged throughout the duration of the study. Sociodemographic data were obtained from each patient as well as clinical data related to smoking status, hypertension or dyslipidaemia. Hypertension was defined as the presence of systolic blood pressure >130 mmHg and/or diastolic blood pressure >85 mmHg, or drug therapy for hypertension. Dyslipidaemia was defined by high-density lipoprotein cholesterol (HDL-C) levels < 40 mg/dL in males or <50 mg/dL in females, triglyceride levels >150 mg/dL or treatment with hypolipidaemic drugs (statins, omega 3 fatty acids and fibrates). The levels of LDL-C considered as dyslipidaemia varied according to the CV risk [80,81].

Physical examination was performed for each participant. Weight (kg) and height (cm) were measured with a weight meter and height rod, respectively. Waist circumference was measured in centimetres with a non-stretchable tape. BMI was calculated as weight (in kg) divided by height in squared meters (kg/m^2^) [82]. Obesity was defined as BMI ≥ 30 kg/m^2^ [82]. Blood samples were obtained in serum and EDTA (ethylenediaminetetraacetic acid) tubes for biochemical and molecular analyses at baseline and at the end of the follow-up period. 

None of the patients discontinued the treatment, and no adverse effects were reported. Four patients developed mild, transient gastrointestinal symptoms during the first 2 weeks of the treatment, which resolved spontaneously after a few days. The high rate of adherence to treatment (100%) was maintained by telephone contact with all the patients during the entire study.

### 4.1. Biochemical Analyses

All samples were obtained after a 14 h overnight fast and then centrifuged within 30 min of collection, in order to make aliquots of both serum and plasma. Serum levels of glucose were determined using a standard glucometer. Glycosylated haemoglobin (HbA1c) was assessed with an immunoassay (DCA Vantage Analyzer, Siemens Healthineers, Erlangen, Germany). Total cholesterol, triglycerides and HDL-C were measured by standard enzymatic–colorimetric methods [83,84,85]; LDL-C was calculated using the Friedewald formula [80].

### 4.2. Extraction of miRNAs and Stem-Loop Real-Time Polymerase Chain Reaction

Total RNA (including small RNA) was extracted from the serum using a NucleoSpin miRNA Plasma kit (Macherey-Nagel, Frankfurt, Germany). The RNA concentration was determined using the Synergy™ HTX Multi-Mode Microplate Reader using Take3 Micro-Volume Plates (BioTek Instruments Inc., Winooski, VT, USA). 

The quantification of miRNAs was carried out using the SYBR Green Real-Time (RT) polymerase chain reaction (PCR) (Thermo-Fisher Scientific, Waltham, MA, USA). Briefly, 1 ng of template RNA was reverse transcribed (in 20 µL) using a stem-loop primer. For the PCR reaction, 1 µL of RT product was used. The PCR was carried out using the Magnetic Induction Cycler PCR detection system (Bio Molecular Systems, Upper Coomera, Australia). Both the RT and PCR reactions were performed in triplicate, in 3 separate experiments. The analysed miRNAs were considered as present when the threshold cycle values were lower than 30. The U6 snRNA was used as the house-keeping gene. The primers for U6 snRNA were forward, GCT TCG GCA CAT ATA CTA AAA T, and reverse, CGC TTC ACG AAT TTG CGT GTC AT. The forward- and reverse-transcribed primers for the different miRNAs are listed in Table 5. The common reverse primer for the PCR of the miRNAs was GTG CGT GTC GTG GAG TC.

There were no differences in the basal levels of miRNAs according to gender or tobacco habits (data not shown).

## 5. Statistical Analysis

Statistical analysis was performed using the statistical software Stata IC 14 (Statacorp LLC, College Station, TX, USA). Continuous variables are expressed as mean ± standard deviation or as the median and interquartile range (IQR), if normally or not normally distributed, respectively. Normality was tested using Shapiro–Wilk tests. Categorical parameters are expressed as percentages. Paired t-tests were used to test differences between baseline and 4-month follow-up variables. Non-parametric Wilcoxon signed-rank tests were performed to compare the differences in the not-normally distributed variables. Correlation analysis was performed using the Spearman rank correlation method. Additionally, multiple regression analysis was performed in order to reveal potential significant independent predictors of changes for specific miRNAs.

## 6. Conclusions

To our knowledge, this is the first report that liraglutide therapy can modulate the serum levels of miRNA-130a, miRNA-27b and miRNA-210 in patients with T2DM. Although the sample size was small, without a placebo group and with a relatively short follow-up period, our findings suggest that liraglutide may exert an epigenetic effect in T2DM patients by regulating miRNAs involved in the maintenance of endothelial cell homeostasis. These liraglutide-induced changes in miRNAs may be implicated in the beneficial cardiometabolic effects of liraglutide, and they may be useful targets for the management of cardiometabolic risk.

## Figures and Tables

**Table 1:** Baseline characteristics of the study population.

Variable	
Age (years), mean ± sd	64.6 ± 8.4
Women, n (%)	9 (36)
Smoking habit, n (%)	10 (40)
Family history of cardiovascular diseases, n (%)	14 (56)
Diabetes duration (years), mean ± sd	9.6 ± 7.1
Systolic blood pressure (mmHg), mean ± sd	128.2 ± 19.3
Diastolic blood pressure (mmHg), mean ± sd	79.4 ± 7.7
Heart rate, bpm	78 ± 9
Hypertension, n (%)	18 (72)
Dyslipidaemia, n (%)	19 (76)
Obesity, n (%)	16 (64)
**Antihypertensive Therapies**	
Beta-blockers, n (%)	12 (48)
Angiotensin-converting enzyme inhibitors, n (%)	7 (28)
Calcium entry blockers, n (%)	7 (28)
Diuretics, n (%)	8 (32)
**Lipid-lowering Drugs**	
Statins, n (%)	13 (52)
Omega-3 fatty acids, n (%)	7 (28)
Fibrates, n (%)	0 (0)
Aspirin use, n (%)	7 (28)

sd: standard deviation.

**Table 2:** Effects of the 4-month liraglutide therapy on cardiometabolic risk parameters.

	Baseline	After 4 Months	*p*-Value
Weight (kg), mean ± sd	78.2 ± 12.4	75 ± 10.9	0.0609 *
BMI (kg/m^2^), mean ± sd	29.2 ± 4.3	28.3 ± 3.5	0.0645 *
Waist circumference (cm), mean ± sd	105.7 ± 10.7	103.2 ± 10.8	0.0894 *
Fasting glucose (mmol/L), median (IQR)	8.4 (3.4)	6.8 (2.4)	0.0052 †
HbA1c (%), median (IQR)	8.3 (0.6)	6.2 (1.5)	0.0009 †
Total cholesterol (mmol/L), mean ± sd	5.0 ± 1.0	4.0 ± 0.7	0.0007 *
Triglycerides (mmol/L), mean ± sd	1.9 ± 1.0	1.5 ± 0.8	0.0478 *
HDL-C (mmol/L), mean ± sd	1.1 ± 0.3	1.2 ± 0.3	0.7659 *
LDL-C (mmol/L), mean ± sd	2.9 ± 1.2	2.2 ± 0.6	0.0150 *

BMI: Body mass index; HbA1c: Glycated haemoglobin; HDL-C: High-density lipoprotein cholesterol; LDL-C: Low-density lipoprotein cholesterol. Values expressed as mean ± standard deviation (sd) or median and interquartile range (IQR). * Paired *t*-test; † Wilcoxon signed-rank test.

**Table 3:** Serum microRNA (miRNA) variation in the study patients.

	Fold Change	*p*-Value *
miRNA-27b	1.73 (7.12)	0.0401
miRNA-130a	1.91 (3.64)	0.0401
miRNA-210	2.09 (11.0)	0.0486

Values represented as the median and interquartile range (IQR). * Wilcoxon signed-rank test.

**Table 4:** Spearman correlation analysis for all patients between changes in specific miRNAs and changes in all the evaluated parameters after 4 months of liraglutide treatment.

	miRNA-27b		miRNA-130a		miRNA-210	
	Correlation Coefficient (r^2^)	*p*-Value	Correlation Coefficient (r^2^)	*p*-Value	Correlation Coefficient (r^2^)	*p*-Value
Weight	0.187	0.4433	0.069	0.7805	0.062	0.7998
BMI	0.179	0.4633	0.066	0.7889	0.072	0.7670
Waist circumference	0.332	0.1643	0.244	0.3146	0.165	0.5009
Fasting glucose	0.072	0.7699	0.169	0.4881	0.008	0.9744
HbA1c	−0.172	0.4813	−0.199	0.4133	−0.373	0.1156
Total cholesterol	0.260	0.2830	0.104	0.6731	0.221	0.3629
Triglycerides	0.098	0.6885	0.317	0.1855	0.001	0.9971
HDL cholesterol	−0.198	0.4159	−0.322	0.1887	−0.333	0.1641
LDL cholesterol	0.256	0.2898	0.220	0.3648	0.408	0.0828

**Table 5:** Primers for miRNA analysis.

miRNA ID	Reverse-Transcribed Primers (48-bp Stem-Loop + 6-bp miRNA-Specific Sequence)	Forward	Reverse (Common Sequence)
Hsa-miRNA-27b	GTCGTATCCAGTGCGTGTCGTGGAGTCGGCAATTGCACTGGATACGACGCAGAA	ATGCTTCACAGTGGCT	GTGCGGTCGTGGAGTC
Hsa-miRNA-130a	GTCGTATCCAGTGCGTGTCGTGGAGTCGGCAATTGCACTGGATACGACATGCCC	ATGCCAGTGCAATGTT	GTGCGGTCGTGGAGTC
Hsa-miRNA-210	GTCGTATCCAGTGCGTGTCGTGGAGTCGGCAATTGCACTGGATACGACTCAGCC	ATGCCTGTGCGTGTGA	GTGCGGTCGTGGAGTC
Common Reverse Primers	GTGCGTGTCGTGGAGTC		

miRNA: MicroRNA; bp: base pairs.
